# Introduction: immunopathology of unresolved tropical diseases

**DOI:** 10.1007/s00281-020-00797-x

**Published:** 2020-06-17

**Authors:** Marcel Tanner

**Affiliations:** 1 Swiss Tropical and Public Health Institute, Basel, Switzerland; 2 University of Basel, Basel, Switzerland

The present issue of Seminars in Immunopathology is motivated by the fact that the diseases of poverty, HIV/AIDS, tuberculosis and malaria, and the neglected tropical diseases (NTDs; [1]) still represent the major burden of ill health in tropical and subtropical areas, particularly in the low- and middle-income countries. The burden of disease as measured by disability adjusted life years (DALYs) of this group of communicable diseases is still massive, as we still lose more than 250 million health life years per year despite major advances made in global public health over the past decades [2]. While the advances in understanding host-pathogen relationships greatly benefited from the well-documented successes in the fields of genomics, proteomics, lipidomics, glycosomics, and the likes, the insight in the pathogen-host crosstalk increases, but we still lack effective vaccines against diseases of poverty and NTDs, and the pipelines for R&D of respective new drugs and treatments are weak, and few new chemical entities are on the horizon. Is there a way forward?

The life cycles of the diseases of poverty and of NTDs are highly virtuous. Rapid sexual and asexual multiplication in sometimes very different niches of the host and vector body based on high antigenic diversity and accompanied by antigenic variation marks the virtuosity and represents mechanisms of survival. The immune system appears fooled by the overload of triggers by enormous numbers of immunogenetic epitopes that do not elicit protective response/effector mechanisms. Hence, the immune system lags constantly behind the pathogen and rather enables survival in the immunized host than its elimination. Once we observe protection or partial protection, we still lack – even with today’s understanding of immune mechanisms and pathways – good correlation of protection. We appear trapped in the scientific inquiry on our way towards effective new tools in the problem of combining diversity and complexity. As it is documented in this fascinating series of innovative reviews contained in the present issue, the combination of diversity and complexity creates the synergies for new solutions, approaches, and tools like vaccines. We therefore see the present issue that immunologically explores host-pathogen relationships, guided by Scott Page’s writing on diversity and complexity “…Diversity plays a different role in a complex system than it does in an equilibrium system, where it often merely produces variation around the mean for performance measures. In complex adaptive systems, diversity makes fundamental contributions to system performance…” [3].

The special issue cannot cover the full spectrum of issues that make up the complexity and diversity of the diseases of poverty and NTDs, but the series of authoritative reviews help us to develop the research and the R&D agenda for the next decade. The review of Alvar et al. [4] introduces us to the field and points at the importance of not only understanding overt pathological processes but also understanding the role of the asymptomatic infections, a feature of NTDs, that govern the host-pathogen relationship and may provide the key to understanding the generation of protective response. This key question is elegantly developed further by the paper of Kaye et al. [5] that examines the complexity of the immune responses and immunopathology in leishmaniosis with regard to the impact on the development of vaccines and also diagnostics. Most recent work in malaria by Schmaler et al. [6] provides innovative insight on the involvement of unconventional T cells in immunity and points at the most promising avenue of research to explore further the functional relevance of the set of unconventional T cells. This line of thinking is taken up and brought to a much broader perspective by the truly innovative contribution of Ramlaho et al. [7] where the complexity and diversity is not only discussed but placed into the realistic and necessary context of how the immune response is modulated by nutrients. Placing our diseases challenges into the context of immune metabolism shows us how we may achieve new tools to control as well as how we can curb unwanted immunological reactions. It is this very insight – combined with decade-long experience – that guides us towards the priority avenues for R&D. The paper of Brazier et al. [8] provides the promising outlook for the development of new vaccines against *Mycobacterium tuberculosis* by assessing the realistic targets for any vaccine development. Following these investigations, Röltgen et al. [9] reviews the broader context of the fascinating evolution of mycobacteria from environmental living styles towards the intracellular style of life but with the special evolutionary, transitory “phases” of *M. ulcerans* and *M. leprae*. Understanding the evolutionary dimensions in relation to our immunological knowledge not only helps us to position a pathogen but also offers clear and coherent approaches towards vaccine developments as well as the public health action when tackling these pathogens. It is finally the comprehensive review by McManus et al. [10] that takes the complex example of schistosomiasis to illustrate how the deep understanding of the immunology in different animal and human schistosomiasis species helps to make a vaccine against schistosomiasis a reality, not only technically but also operationally for the benefit of the human and animal populations concerned.

We truly hope that the present issue does not leave you, being a scientists or a public health specialist, at despair. There is a way forward, and we hope you see evolve what initially appeared as a non-connected set of fragments and uncoordinated patchwork of complexity and diversity, an evolution to a well-profiled and unique, shining quilt of a more integrated view forward on how we can tackle the initially called “unresolved tropical diseases” problems by using the immunological and metabolic insights to structure and propose an agenda for priority research and development in order to achieve solutions for better health and well-being.
